# Obesity, dyslipidaemia and candidate gene polymorphisms: a cross-sectional study among the Liangmai and Mizo tribes of Manipur, India

**DOI:** 10.1080/07853890.2021.1969034

**Published:** 2021-08-20

**Authors:** Somorjit Singh Ningombam, Masan Kambo Newmei, Varhlun Chhungi, Prakash Ranjan Mondal, Naorem Kiranmala Devi, Kallur Nava Saraswathy

**Affiliations:** a Laboratory Oncology Unit, Dr. B.R.A. Institute Rotary Cancer Hospital, All India Institute of Medical Sciences, New Delhi, India; b Department of Neurology, All India Institute of Medical Sciences, New Delhi, India; c National Family Health Survey-5, ICMR-National Institute of Malaria Research, New Delhi, India; d Department of Anthropology, Laboratory of Biological and Molecular Anthropology, University of Delhi, New Delhi, India

**Keywords:** Cardiovascular diseases, genetic association variation, tribes, North-East India

## Abstract

**Background:**

The prevalence of obesity and dyslipidaemia was observed to be increased among the tribal populations, due to globalization.

**Materials and methods:**

In the present study, data on demographic, somatometric and blood samples were collected from 613 participants of both sex, age 18–60 years, further lipid profiling and genotyping was executed. Multifactor dimensionality reduction (MDR) software was used for gene–gene interactions analysis.

**Results:**

Significantly differences were observed with respect to the general characteristic and selected gene polymorphisms in both the tribes. Among the Liangmai tribe, *MC4R* gene was found to pose significant decreased risk for waist–height ratio (WHtR) (OR = 0.56; 95% confidence interval (CI)= 0.32–0.99; *p* value = .04) and HDL (OR = 0.58; 95% CI = 0.36–0.92; *p* value = .02). Similar trends of significant decreased risk (OR = 0.39; 95% CI = 0.20–0.76; *p* value=.006) for BMI were observed among the Mizo tribe. The gene–gene interaction revealed the combined model of *FTO*+*MC4R* genes shows an increased risk for BMI in both the tribes. The independent significant increased risk posed by *FTO* gene was moderated by interaction with *MC4R* gene.

**Conclusions:**

The observed differences can possibly attribute to both their respective ancestries resulting in different gene pools and the physical environment. The results of the study highlight the importance of gene–gene and gene–environment interactions in adverse phenotype groups.

**KEY MESSAGE**
- Among the tribal population, the prevalence of obesity and dyslipidaemia has been increased.
- Differential distribution and associations of selected markers hint towards differential genetic architecture in these populations.
- *MC4R* rs17782313 polymorphism was found to show a significantly decreased risk for WHtR and low HDL among the Liangmai tribe and BMI among the Mizo tribe.
- Significant increased risk posed by *FTO* rs9939609 gene polymorphism was moderated by the interaction with *MC4R* rs17782313.

## 1. Introduction

Overweight and obesity are defined as the abnormal or excessive fat accumulation that may impair health [1]. The prevalence of obesity is still increasing in both developed and developing countries [2]. The distribution of overweight and obesity varies widely among the different population groups across the globe including India [2]. In Indian context where diversity exists at every level, obesity has been reported to be as low as 1.50% to as high as 45.6% [3,4]. Of the eight North-eastern states of India, Sikkim is reported to have the highest prevalence of obesity (37.8% – urban, 26.4% – rural) [4]. In Manipur, the highest prevalence of overweight and obesity till date is reported to be as high as 63% (overweight) and 14% (obese) for the general population and 27.1% of Tangkhul Naga tribes [5–7]. The differences in the distribution of overweight and obesity in the Indian context can conveniently be attributed not only to lifestyle factors (i.e. dietary habits and physical activity), but also to genetic predisposition owing to their ancestry [8].

Dyslipidaemia is defined as the derangement of one or more lipoproteins that is increased levels of serum cholesterol or serum triglycerides (TGs) or low-density lipoprotein cholesterol (LDL-C) or decreased levels of high-density lipoprotein cholesterol (HDL-C) [9]. Indian population have different dyslipidaemia patterns compared to other populations, which is signified by lower HDL-C, increased TG and increased small dense LDL cholesterol level [10]. Both the co-morbidities, i.e. obesity and dyslipidaemia are the most important factors that epitomize an increased risk for cardiovascular adversities such as type-2 diabetes, hypertension, stroke, etc. and some forms of cancer [2,11]. Various risk factors, i.e. biological, social and behavioural are responsible for such co-morbidities. Further these factors are influenced by globalization, demographic changes, socio-political situations, education and cultural norms [12].

Whether it is dyslipidaemia or obesity, it refers to the accumulation of fat. This accumulation results when there is a blockage in the metabolic pathways, all the steps of which are controlled by genes. The differences in the distribution of overweight and obesity in the Indian context can conveniently be attributed not only to lifestyle factors (i.e. dietary habits and physical activity), but also to genetic predisposition owing to their ancestry [9].

The mutation in the genes is likely to lead to disturbances in the pathway resulting to dyslipidaemia and obesity. Till date, 97 genetic loci have been identified through GWAS that are robustly associated with obesity. Most associated genes that are reported to be associated with obesity are – *FTO* rs9939609 and *MC4R* rs17782313, recent studies also reported *ACE I/D* rs4646994 and *MTHFR C677T* rs1801133 gene polymorphisms to be associated with obesity specifically in Indian population [13,14]. These selected candidate gene polymorphisms of obesity were also found to be associated with dyslipidaemia [15]. However, inconsistent results have been reported on distribution of obesity and dyslipidaemia and its associations with the selected genetic polymorphism with non-European ancestry, specifically African Americans, South Asians and Southeast Asians. Moreover, studies on such obesity related genetic polymorphisms have not been reported from any of the North-East Indian (tribal) populations that are supposed to have South-East Asian ancestry.

Thus, the major aim of the present study is to understand the distribution of various obesity, dyslipidaemia and genetic variables in the two selected populations groups residing in two different environmental settings. The study further attempts to see the associations between the selected gene polymorphisms on obesity and dyslipidaemia variables.

## 2. Materials and methods

### 2.1. Study population

The field area of the study was Manipur, North-East state of India. A cross-sectional study was conducted among two tribal populations, namely, Liangmai which belongs to a Naga tribe and Mizo which belongs to the Kuki-Chin-Mizo tribal group. Liangmai tribe was found to spread across Tamenglong and Senapati district while Mizo tribe was found to predominantly reside in Churachandpur district. Both the tribes have been reported to have South-East Asian Ancestry and a close affinity with Han Chinese [16]. Literature about the origin of both the tribes suggests that Liangmai was original habitant of the hills slopes of Manipur while Mizo had gradually migrated from Mizoram [17].

Participants were recruited from both sexes with age ranging from 18 to 60 years after obtaining a pre-informed consent from each participant. A total of 613 participant (342 Liangmai and 271 Mizo) unrelated up to first cousin were recruited from both the tribes. A detailed information of all the participants on demographic (name, age, sex, place of birth, education and occupation) and somatometric (height, weight, waist circumference and hip circumference) were taken using pre-tested interview schedules and standard techniques, respectively. An over-night fasting intravenous blood (5 ml) was also collected by a trained technician for biochemical and molecular works. However, the data on somatometric variables, physiological and biochemical variables were not available for all the recruited participants due to dropout during interview, measurements and blood sample collection. The study was approved by the Departmental Ethics Committee, Department of Anthropology, University of Delhi, India.

### 2.2. Somatometric measurements and sample classification

The classification of body mass index (BMI) was performed according to Asia Pacific population criteria, i.e. underweight (<18.5 kg/m^2^), normal weight (18.5–22.99 kg/m^2^), overweight (23–25 kg/m^2^) and obese (>25 kg/m^2^) [18]. Waist circumference (cm) was measured as the least circumference between the lower ribs and the iliac crest. Waist circumference <90 cm for males and <80 cm for female was taken as normal [19]. Hip circumference was measured at the buttock yielding the maximum circumference. Waist–hip ratio was calculated using the formula waist (cm) divided by hip (cm), i.e. (W/H) [20]. The circumferences were measured using a non-expendable steel tape. Normal WHR calculated as was classified as <0.90 (males), <0.80 (females) [19]. Waist–height ratio (WHtR) was waist (cm) divided by height (m) and the cut-off values were taken as <0.40 (underweight), ≥0.40 to <0.50 (normal weight), ≥0.50 to <0.60 (high risk) and ≥0.60 (morbidly high) [21].

### 2.3. Lipid measurement and sample classification

According to the manufacturing protocol (Randox, Kearneysville, WV), lipid profiling was done by using spectrophotometer. Normal cut-off value for different biochemical variables was considered as total cholesterol (100–200 mg/dl), TG (50–150 mg/dl), HDL-cholesterol (male >40 mg/dl; female >50 mg/dl), LDL cholesterol (up to 130 mg/dl) and VLDL (10–30 mg/dl) [22]. The values of LDL cholesterol and VLDL cholesterol were calculated using Friedwald and Fredrikson’s formula.

### 2.4. Polymerase chain reaction (PCR) and genotyping

DNA was extracted from the collected blood using salting-out method [23]. Genotyping of *MC4R* rs17782313*, MTHFR C677T* rs1801133 gene polymorphisms was carried out by PCR-RFLP method. For *ACE I/D* polymorphism, allelic specific PCR was performed to understand the genetic polymorphism. The PCR was carried out using the thermocycler (C-1000 Touch^TM^, Bio-Rad, Hercules, CA) with their specific primers, protocols and restriction enzymes (Supplementary Table 1).

### 2.5. Statistical analysis

Mann–Whitney’s test was performed to assess the differences in the median values of the continuous variables (age, BMI, WHR, WHtR, TC, TG, HDL, LDL, VLDL) between the two populations. Chi-square (*χ*^2^) test was used to see the difference in the distribution of genotypes and categorical variables (sex, occupation, literacy) in the studied population groups. Normality was checked by using Shapiro–Wilk’s test. Binary logistic regression was used for analysing the odds ratio (OR) with 95% confidence interval (CI) between the somatometric, lipids and selected genetic variables. Individuals with abnormal (high) obesity variables (BMI, WC, WHR and WHtR) and high lipid variables (TC, TG, HDL, LDL, VLDL) were considered as cases and individuals with normal obesity and dyslipidaemia are considered as controls for association analysis. The distribution of various general characteristic, i.e. age, sex, education, occupation, tobacco consumption and alcohol consumption in cases and controls were analysed using *χ*^2^ (Supplementary Tables 2–10), and those variables with significant *p* value were adjusted in their respective association analysis.

**Table 2.** Distribution of *FTO rs9939609*, *MC4R* rs17782313, *ACE I/D* rs4646994, *MTHFR C677T* rs1801133 genes polymorphism among Liangmai and Mizo tribes of Manipur.

Genetic markers	Genotypes	Liangmai			Mizo			Total *χ*^2^ *p* value
		Observed frequency *N* (%)	Expected frequency *N* (%)	*χ*^2^*p* value	Observed Frequency *N* (%)	Expected Frequency *N* (%)	*χ*^2^*p* value	
*FTO* rs9939609	TT TA AA	274 (80.1) 68 (19.88) 0 T-0.90 A-0.10	277.4(81.1) 61.2 (17.9) 3.4 (0.98)	4.17 (**.04**)*	180 (68.44) 81 (30.79) 2 (0.76) T-0.84 A-0.16	184.8 (70.29) 71.2 (27.09) 6.8 (2.61)	4.91 (**.02**)*	.006*
*MC4R* rs17782313	TT CT CC	180 (55.04) 134 (40.97) 13 (3.97) T-0.76 C-0.24	186.6 (57.06) 120.8 (36.96) 19.6 (5.98)	3.87 (**.04**)*	187 (78.51) 48 (20.16) 3 (1.26) T-0.89 C-0.11	187.06(78.59) 47.87 (20.11) 3.06 (1.28)	0.001 (.96)	<.001*
*ACE* rs4646994	II ID DD	73 (21.53) 179 (52.8) 88 (25.95) I-0.48 D-0.52	77.67 (22.91) 169.7 (50.05) 92.67 (27.33)	1.03 (.31)	114 (42.06) 96 (35.42) 61 (32.50) I-0.6 D-0.4	96.84 (35.73) 130.32 (48.1) 43.84 (16.17)	18.79 (<**.001**)*	<.001*
*MTHFR* *C677T* rs1801133	CC CT TT	223 (77.2) 58 (20.1) 8 (2.7) C-0.87 T-0.13	219.7 (76.1) 64.5 (22.3) 4.74 (1.6)	2.96 (.08)	196 (85.6) 33 (14.4) 0 C-0.93 T-0.07	197.2 (86.1) 30.6 (13.4) 1.19 (0.51)	1.38 (.23)	.02*

* *p* Value <.05 is statistically significant.

**Table 3.** Odds ratio analysis of *FTO* rs9939609, *MC4R* rs17782313, *ACE I/D* rs4646994 and *MTHFR C677T* rs1801133 polymorphisms with somatometric and dyslipidaemia variables among the Liangmai and Mizo tribes of Manipur.

Population	Variables	*FTO* rs9939609			*MC4R* rs17782313			*ACE I/D* rs4646994			*MTHFR C677T* rs1801133		
		OR	95% CI	*p* Value	OR	95% CI	*p* Value	OR	95% CI	*p* Value	OR	95% CI	*p* Value
Liangmai	BMI	1.9	1.07–3.6	.02*	0.87	0.54–1.4	.59	1.7	0.67–2.0	.57	0.93	0.50–1.7	.81
	WC	1.73	0.93–3.2	.10	0.64	0.40–1.03	.06	0.81	0.46–1.4	.47	0.99	0.54–1.8	.98
	WHR	1.32	0.42–4.3	.62	0.43	0.4–4.4	.48	0.08	0.002–2.9	.17	0.37	0.12–1.1	.07
	WHtR	1.57	0.74–3.3	.23	0.56	0.32–0.99	.04*	1.2	0.65–2.4	.48	0.98	0.47–2.0	.96
	TC	0.65	0.36–1.17	.15	1.04	0.65–1.66	.85	1.01	0.58–1.73	.98	0.84	0.47–1.51	.57
	TG	0.58	0.32–1.03	.06	0.85	0.54–1.33	.49	0.61	0.36–1.03	.06	0.90	0.51–1.58	.71
	HDL	1.04	0.60–1.8	.86	0.58	0.36–0.92	.02*	0.81	0.47–1.41	.46	0.92	0.52–1.62	.79
	LDL	0.82	0.44–1.5	.52	1.06	0.64–1.75	.81	0.96	0.53–1.71	.89	0.95	0.51–1.74	.86
	VLDL	0.58	0.32–1.03	.06	0.85	0.54–1.33	.49	0.61	0.36–1.03	.06	0.90	0.51–1.58	.71
Mizo	BMI	1.5	0.85–2.8	.14	0.39	0.20–0.76	.006*	0.63	0.36–1.1	.10	0.56	0.25–1.2	.15
	WC	0.88	0.46–1.6	.71	0.91	0.43–1.9	.81	0.97	0.54–107	.93	0.94	0.40–2.2	.89
	WHR	1.73	0.72–4.1	.21	0.94	0.36–2.4	.90	0.58	0.26–1.2	.18	0.81	0.26–2.4	.70
	WHtR	1.97	0.97–3.9	.05*	1.2	0.54–2.6	.64	0.58	0.30–1.0	.09	1.93	0.66–5.6	.22
	TC	0.91	0.53–1.56	.73	0.64	0.34–1.22	.17	1.25	0.75–2.08	.37	0.62	0.29–1.34	.23
	TG	1.42	0.84–2.4	.18	1.51	0.81–2.8	.19	0.94	0.58–1.53	.81	1.37	0.65–2.8	.40
	HDL	1.01	0.54–1.85	.98	0.80	0.38–1.7	.57	0.94	0.54–1.66	.85	0.93	0.39–2.2	.87
	LDL	0.74	0.43–1.27	.28	0.72	0.38–1.36	.32	1.06	0.64–1.76	.79	0.63	0.28–1.36	.24
	VLDL	1.42	0.84–2.4	.18	1.51	0.81–2.82	.19	0.94	0.58–1.53	.81	1.37	0.65–2.8	.40

BMI: body mass index; WC: waist circumference; WHR: waist to hip ratio; WHtR: waist to height ratio; TC: total cholesterol; TG: triglycerides; HDL: high density lipoprotein; LDL: low-density lipoprotein; VLDL: very low-density lipoprotein; OR: odds ratio-adjusted for confounders.

* *p* Value <.05 is statistically significant.

**Table 4.** Genetic interaction results between *FTO* rs9939609, *MC4R* rs17782313, *ACE I/D* rs4646994 and *MTHFR C677T* rs1801133 for somatometric and dyslipidaemia variables using generalized multifactor dimensionality reduction (GMDR).

Variables	Liangmai				Mizo			
	Model	TBA	CVC	*p* Value	Model	TBA	CVC	*p* Value
BMI	*FTO*	0.495	7/10	.15	*MC4R*	0.585	10/10	.004*
	*FTO, MC4R*	0.505	8/10	.01*	*FTO, MC4R*	0.624	10/10	<.001*
	*FTO, MC4R, MTHFR*	0.509	5/10	<.001*	*FTO, MC4R, ACE*	0.619	10/10	<.001*
WC	*MC4R*	0.584	10/10	.007*	*FTO*	0.539	10/10	.19
	*FTO, MC4R*	0.529	7/10	.001*	*MTHFR, ACE*	0.417	4/10	.04*
	*MC4R, MTHFR, ACE*	0.509	7/10	<.001*	*FTO, MC4R, ACE*	0.385	5/10	.05*
WHR	*ACE*	0.450	5/10	.07*	*FTO*	0.563	9/10	.05*
	*MC4R, ACE*	0.589	9/10	.003*	*FTO, ACE*	0.447	5/10	.03*
	*MC4R, MTHFR, ACE*	0.452	6/10	<.001*	*FTO, MC4R, ACE*	0.525	10/10	.002*
WHtR	*MC4R*	0.572	10/10	.01*	*FTO*	0.603	10/10	.01*
	*FTO, MC4R*	0.564	7/10	.003*	*FTO, ACE*	0.536	6/10	.01*
	*FTO, MC4R, ACE*	0.533	9/10	<.001*	*FTO, MTHFR, ACE*	0.525	10/10	.002*
TC	*FTO*	0.525	10/10	.316	*MC4R*	0.559	10/10	.05*
	*MC4R, ACE*	0.458	6/10	.172	*MC4R, ACE*	0.549	9/10	.03*
	*FTO, MC4R, MTHFR*	0.459	6/10	.071	*MC4R, MTHFR, ACE*	0.492	5/10	.031*
TG	*FTO*	0.504	8/10	.072	*FTO*	0.569	10/10	.042*
	*FTO, MTHFR*	0.563	10/10	.013*	*FTO, MC4R*	0.516	8/10	.042*
	*FTO, MC4R, MTHFR*	0.543	10/10	.005*	*FTO, MC4R, ACE*	0.502	7/10	.019*
HDL	*MC4R*	0.570	10/10	.018*	*FTO*	0.554	10/10	.145
	*FTO, MC4R*	0.543	8/10	.018*	*FTO, MC4R*	0.505	6/10	.046*
	*FTO, MC4R, MTHFR*	0.553	5/10	.010*	*FTO, MC4R, MTHFR*	0.497	9/10	.012*
LDL	*MC4R*	0.358	5/10	.909	*MC4R*	0.529	9/10	.09
	*FTO, MTHFR*	0.346	4/10	.664	*FTO, MC4R*	0.466	6/10	.09
	*FTO, MC4R, MTHFR*	0.3487	5/10	.393	*FTO, MC4R, MTHFR*	0.552	10/10	.05*
VLDL	*FTO*	0.504	8/10	.072*	*FTO*	0.569	10/10	.042*
	*FTO, MTHFR*	0.563	10/10	.013*	*FTO, MC4R*	0.516	8/10	.042*
	*FTO, MC4R, MTHFR*	0.543	10/10	.005*	*FTO, MC4R, ACE*	0.502	7/10	.019*

BMI: body mass index; WC: waist circumference; WHR: waist height ratio; WHtR: waist height ratio; TC: total cholesterol; TG: triglycerides; HDL: high density lipoprotein; LDL: low-density lipoprotein; VLDL: very low-density lipoprotein; TBA: testing balance accuracy; CVC: cross validation consistency.

* *p* Value <.05 is statistically significant.

**Table 5.** Intra-genetic epistatic interaction between the potential genetic loci among the Liangmai and Mizo tribes.

Variables	Liangmai				Mizo			
	Best model	OR	95% CI	*p* Value	Best model	OR	95% CI	*p* Value
BMI	*FTO, MC4R*	1.94	0.78–4.8	.15	*FTO, MC4R*	2.16	0.45–10.3	.33
WC	*MC4R*	0.64	0.40–1.03	.06	*FTO*	0.88	0.46–1.6	.71
WHR	*MC4R, ACE*	0.55	0.20–1.5	.25	*FTO, MC4R, ACE*	0.80	0.08–8.0	.35
WHtR	*MC4R*	0.56	0.32–0.99	.04*	*FTO*	1.97	0.97–3.9	.05
TC	*FTO*	0.65	0.36–1.17	.15	*MC4R*	0.64	0.34–1.22	.17
TG	*FTO, MTHFR*	1.541	0.48–4.9	.46	*FTO*	1.42	0.84–2.4	.18
HDL	*MC4R*	0.585	0.36–0.92	.02*	*FTO*	1.006	0.34–1.85	.93
LDL	*MC4R*	1.06	0.64–1.75	.81	*FTO, MC4R, MTHFR*	0.386	0.07–2.05	.26
VLDL	*FTO, MTHFR*	1.541	0.48–4.9	.46	*FTO*	1.42	0.84–2.4	.18

BMI: body mass index; WC: waist circumference; WHR: waist to hip ratio; WHtR: waist to height ratio; TC: total cholesterol; TG: triglycerides; HDL: high density lipoprotein; LDL: low-density lipoprotein; VLDL: very low-density lipoprotein; OR: odd ratio-adjusted for confounders.

* *p* Value <.05 is statistically significant.

All analyses were performed using the Statistical Package for the Social Sciences (SPSS, for window version 20.0, Chicago, IL). Statistical significance was taken at *p* value <.05 for all the comparisons.

Multifactor dimensionality reduction (MDR) software was used for gene–gene interactions analysis [24]. It is a non-parametric and model-free method that transforms a high-multilocus model to a single dimension model. The MDR algorithm starts with a 10-fold cross-validation for each possible set of factors to determine the best set of factors. In this process, datasets are divided into a training part (9/10 of the data) and testing part (1/10 of the data). Further, the selection of the best model was done by selecting the model which has the smallest prediction error and/or the largest cross-validation consistency.

## 3. Results

The two selected tribal populations were found to be significantly different with respect to all the selected general characteristics. Mizo tribe is found to be older in age than Liangmai tribe but also experience higher exposure to tobacco consumption, literacy and sedentary lifestyle (Table 1).

**Table 1.** Distribution of general characteristics among Liangmai and Mizo tribes of Manipur.

Variables	Liangmai, *N* (%)	Mizo, *N* (%)	*p* Value
Age (median (IQR))	40 (31.25–49.75)	45 (33–56)	<.001*
Alcohol consumption			
No	55 (77.5)	268 (66.2)	.06
Yes	16 (22.5)	137 (33.8)	
Tobacco consumption			
No	48 (68.6)	134 (33.1)	<.001*
Yes	22 (31.4)	271 (66.9)	
Education			
Illiterate	57 (16.1)	0	<.001*
Literate	297 (83.9)	405 (100)	
Self-reported physical activity			
Sedentary	66 (29.2)	198 (48.9)	<.001*
Active	160 (70.8)	207 (51.1)	

* *p* Value <.05 is statistically significant.

All the selected genetic loci were found to be polymorphic in the presently studied populations. Both the tribal populations were found to deviate from the Hardy-Weinberg equilibrium (HWE), with respect to the *FTO* rs9939609 polymorphism. *MC4R* rs17782313 polymorphism was found to follow the HWE among the Mizo tribe, whereas *ACE I/D* rs4646994 polymorphism was found to follow HWE among the Liangmai tribe. For *MC4R* and *ACE I/D* polymorphism, the minor allele frequency was found to be lower among the Mizo tribe, i.e. C-0.11 and D-0.4, respectively as compared to that of the Liangmai tribe, i.e. C-0.24 and D-0.52, respectively (Table 2). Interestingly, both the population were found to follow HWE with respect to the *MTHFR C677T* rs1801133 polymorphism with the minor allele frequency of T-0.13 and T-0.07 among the Liangmai and Mizo tribe, respectively (Table 2).

The relative risk analysis for of *FTO*, *MC4R*, *ACE* and *MTHFR* gene polymorphism with obesity and dyslipidaemia is performed independently in both the studied populations. Odds ratio analysis revealed that minor allele of *FTO* rs9939609 polymorphism was found to pose significant increased risk for BMI and WHtR, among the Liangmai and Mizo tribe, after adjusting for their respective confounders. Further in Liangmai tribe, minor allele of *FTO* rs9939609 polymorphism was found to pose decreasing odds (OR = 0.58; 95% CI = 0.32–1.03; *p* value=.06) for TG and VLDL with a suggestive *p* value after controlling their respective confounders (Table 3) (Supplementary Tables 7 and 10). Whereas among the Mizo tribe, none of the lipid variables were found to have any association with *FTO* rs9939609 gene polymorphism (Table 3). In case of Liangmai tribe, minor C allele of *MC4R* was found to pose significant decreased risk for WHtR (OR = 0.56; CI = 0.32–0.99; *p* value=.04) and HDL (OR = 0.58; CI = 0.36–0.92; *p* value=.02) after controlling their respective confounders (Table 3) (Supplementary Tables 5 and 8). Similar trends of significant decreased risk (OR = 0.39; CI = 0.20–0.76; *p* value=.006) for BMI were observed among the Mizo tribe after adjusting for age (Table 3) (Supplementary Table 2). Further, remaining somatometric and lipid variables were not found to show any association with the *MC4R* gene polymorphism. There were no significant associations between the selected *ACE I/D* and *MTHFR* genetic polymorphisms with respect to the somatometric and lipid variables, after adjusting their respective confounders in both the studied populations. However, among the Liangmai tribe, *ACE I/D* polymorphism showed decreased risk for TG and VLDL, and MTHFR polymorphism for WHR among the Liangmai tribe with suggestive *p* value, after controlling the confounders (Table 3). All the confounders that have adjusted for the present association studies were given as supplementary file with their respective phenotypes (Supplementary Tables 2–10).

The best model for gene–gene interactions analysis was selected based on the highest value of cross validation consistency (CVC) and test balanced accuracy (TBA) according to MDR analysis (Table 4). In both the tribes, the best models for BMI, WC, WHR, WHtR, TC, TG, HDL, LDL, VLDL were observed as *FTO*+*MC4R* (CVC = 8/10; *p*=.01), *MC4R* (CVC = 10/10; *p*=.007), *MC4R*+*ACE* (CVC = 9/10; *p*=.003), *MC4R* (CVC = 10/10; *p*=.01), *FTO* (CVC = 10/10; *p*=.01), *FTO*+*MTHFR* (CVC = 10/10; *p*=.90), *MC4R* (CVC = 10/10; *p*=.01), *MC4R* (CVC = 5/10; *p*=.90) and *FTO*+*MTHFR* (CVC = 10/10; *p*=.01) for Liangmai tribe and *FTO*+*MC4R* (CVC = 10/10; *p*= <.001), *FTO* (CVC = 10/10; *p*=.19), *FTO*+*MC4R*+*ACE* (CVC = 10/10; *p*=.002), *FTO* (CVC = 10/10; *p*=.01), *MC4R* (CVC = 10/10; *p*=.05), *FTO* (CVC = 10/10; *p*=.04), *FTO* (CVC = 10/10; *p*=.14), *FTO*+*MC4R*+*MTHFR* (CVC = 10/10; *p*=.05) and *FTO* (CVC = 10/10; *p*=.04) for Mizo tribe, respectively (Table 4).

The epistatic intra-genetic interaction between the potential genetic loci showed that the combined model of FTO and MC4R genes shown an increased risk for BMI in both the tribal populations but not statistically significant (OR = 1.94; 95% CI = 0.78–4.8; *p* value=.15). Only the *MC4R* rs17782313 was seen to pose significant reduced risk for WHtR and HDL, and reduced risk for WC with a suggestive *p* value among the Liangmai tribe (Table 5). The combined model of *FTO* and *MTHFR* showed increased risk for TG and VLDL, but not statistically significant among the Liangmai tribe. Whereas in case of Mizo tribe, *FTO* independently poses increased risk for WHtR, TG, HDL and VLDL, except for WC showing reduced risk; however, all the results were not found to be statistically significant (Table 5). Finally, the genetic model of *FTO*, *MC4R*, *ACE* and *FTO*, *MC4R*, *MTHFR* shows reducing risk for WHR and LDL, respectively, but not statistically significant.

## 4. Discussion

An increase in the adverse lifestyles such as smoking and tobacco consumption, intake of unhealthy diet and sedentary lifestyle is directly associated with the increased prevalence of hypercholesterolaemia and cardiovascular diseases [25]. In the present study, factors responsible for the increase in non-communicable diseases such as higher age, tobacco consumption, alcohol consumption and a sedentary lifestyle were found to be significantly higher among the Mizo tribe as compared to that of Liangmai tribe. These could be the major contributing factors in the higher prevalence of all dyslipidaemia variables among the Mizo tribe.

The possible reasons for the deviation from HWE for *FTO* rs9939609, *MC4R* rs17782313 and *ACE* rs4646994 polymorphisms could be the over representation of heterozygote among the Liangmai tribe and over representation of homozygote among the Mizo tribe. Interestingly, both the populations were found to follow HWE with respect to the *MTHFR* C677T polymorphism with the minor allele frequency of T-0.13 and T-0.07 among the Liangmai and Mizo tribe, respectively. Significant differences observed between the two tribes with respect to all the selected gene polymorphism with respect to the entire selected gene polymorphism hint towards the differential genetic architecture of these two populations which could be attributed to either differential ancestries or some sort of differential selection pressure on the studied genetic polymorphisms in the two population groups. A study conducted by Kameih et al. on the origin and migratory history of tribal populations of Manipur, supported the present finding where genomic affinities and differentiation were observed among the Naga and Kuki tribal populations [26]. Further, these genes behaved differently in the present studied populations inhabited in the same geographical area indicating that their impact on health also is likely to be different.

With respect to genetic association of the four selected gene polymorphisms, *FTO* rs9939609 and *MC4R* rs17782313 were found to be associated with obesity. Earlier, the author of the present study already reported the association of the *FTO* rs9939609 with BMI among the Liangmai tribe and central obesity in term of WHtR in Mizo tribe [27], in the present study, data of *FTO* gene were used for the purpose of gene–gene interaction analysis. *FTO* rs9939609 is found to be associated with TG and VLDL among the Liangmai tribe with a suggestive *p* value. The present finding was supported by Zhou et al., where *FTO* rs9939609 was found to be significantly associated with metabolic syndrome [28]. The fact that A allele is posing an increased risk for high BMI (general obesity), TG and VLDL in Liangmai tribe and increased risk for WHtR (central obesity) in Mizo tribe, suggests a differential association of the gene polymorphism in these two different population groups. According to GWAS, melanocortin 4 receptor (*MC4R*) gene was reported to the second strongest gene in the causes of obesity next to *FTO* gene [29]. The majority of the studies reported significant increased association with obesity and *MC4R* rs17782313; however, discrepancies were observed, especially among the East Asian and Africans [30]. In the present study, *MC4R* rs17782313 polymorphism was found to show significant decreased risk for WHtR and low HDL among the Liangmai tribe and BMI among Mizo tribe, respectively. Further, the present observations were supported by various findings which show decreasing risk of *MC4R* rs17782313 polymorphism with WC, BMI, HDL in European and Asian population, which finally revealed that *MC4R* genes not only induced obesity and dyslipidaemia, it also reduced their risks in some population groups [31]. The discrepancy in the results might be because of the differences in genetic architecture and environmental background of the studied population groups.

Besides, two genes (*ACE I/D* and *MTHFR C677T*) polymorphisms are not found to have any influence on obesity. The findings of the present study were supported by the previous studies, that *ACE* genotype was not associated with both in general (BMI) and central obesity (WC and WHR) [32]. With respect to *ACE I/D* polymorphism, II genotype was reported to be associated with lower serum level of TC, LDL, non-HDL [33]; however, opposite result was observed in the present study where DD genotype showed a negative association with TG and VLDL among the Liangmai tribe with suggestive *p* value. The present finding was supported by Garatachea et al. where DD genotype and D allele have significant advantages to reach exceptional longevity [34]. For *MTHFR C677T*, a longitudinal Copenhagen City Heart Study supported the present finding, where TT genotype of the *MTHFR* gene was not found to be associated with obesity or BMI. Albeit, population with higher socio-economic status and good nutrition lead to the survival advantages of TT genotype of *MTHFR* gene [35]. Mizo tribe is mainly inhabited in Churachandpur district of Manipur State, they experience good socio-economic status, as an evidence 9.80% of establishment and 9.66% of employed was contributed in the whole state as compared to Tamenglong district where Liangmai tribe where inhabited [36]. Interestingly, among the Mizo tribe, TT genotype individuals could not survive even though they experience good nutrition and higher socio-economic status compared to Liangmai tribe. This could be because of the higher exposure of this population to the bad environment in terms of lifestyle, i.e. higher consumption of alcohol and tobacco, sedentary lifestyle and older ages lead to disturb the pathways of *ACE* and *MTHFR* genes.

Generally, the combined effect of *FTO* rs9939609 and *MC4R* rs17782313 was significantly associated with obesity in various populations [37,38]. In another view, individuals who have mutant allele of both genes may be neutral towards obesity, because the positive effect of the *FTO* rs9939609 gene polymorphism is reduced by the *MC4R* rs17782313. The above hypothesis seems to prove true by the present study, among Liangmai tribe, where there is a significant increased risk posed by *FTO* rs9939609 gene polymorphism was moderated by the interaction with *MC4R* rs17782313 (and the statistical significance was lost). On the contrary, among Mizo, the risk caused by the interaction of *FTO* rs9939609 and *MC4R* rs17782313 for obesity was enhanced, albeit with no statistical significance. Further, after controlling for the interaction effect, only *MC4R* rs17782313 was seen to confer risk for obesity in terms of high WHtR among Liangmai whereas *FTO* rs9939609 was seen to pose significantly reduced risk for WHtR. *ACE,* and *MTHFR* were not found to play any pathogenic or additive effect in the causation of obesity (general or central) in any of the studied population. However, the role of these polymorphisms in addition to the pathogenic role of *FTO* and *MC4R* gene mutations cannot be completely ruled out.

The gene–gene interactions indicated that the selected gene polymorphisms may have an additive or dipping effect on obesity and dyslipidaemia in both Liangmai and Mizo populations. In spite of similar ancestry, the interaction was seen in one population group while it was not seen in another group. Such gene–gene interactions, thus become important in understanding the complex phenotypes such as obesity where population structure, dietary habits, physical environment and biological environment may play an important role.

## 5. Conclusions

The two studied populations have differential distribution not only in the general characteristics, obesity and dyslipidaemia but also in terms of genotypic distribution of the selected gene polymorphisms. Even the association between the genetic polymorphisms seems to be different in the two selected populations. These observed differences can possibly attribute to both their respective ancestries resulting in different gene pools but also the physical environment where Mizo’s reside relatively at higher altitude as compared to that of the Liangmai tribe. The results of present study highlight the importance of gene–gene and gene–environment interactions in different population groups. Hence, the genetic associations studies should be analysed, understood and interpreted in light of various environmental factors.

## Supplementary Material

Supplemental Material

## Data Availability

The data that support the findings of this study are available upon reasonable request.
