# Supplementary material for: Obesity, dyslipidaemia and candidate gene polymorphisms: a cross-sectional study among the Liangmai and Mizo tribes of Manipur, India
Source: Ann Med. 2021 Aug 20;53(1):1439–47. doi: 10.1080/07853890.2021.1969034 (PMC8381916; doi:10.1080/07853890.2021.1969034)
Supplement: Supplemental Material [file IANN_A_1969034_SM7055.docx]

**Summary of supplementary files:**

Supplementary Table 1, explained the primer sequences and PCR conditions of the selected genetic polymorphisms with their specific restriction enzymes. Supplementary table 2-10 shown the General characteristics of cases and controls with respect to phenotypes among the Liangmai and Mizo tribes to find out the confounders for association studies.

Supplementary Table 1: Primer sequences and PCR conditions of the selected genetic polymorphisms with their specific restriction enzymes.

| **Sl no.** | **Amplified DNA regions** | **Primer type** | **Primer sequence (5’-3’)** | **References** | **PCR conditions** | **Restriction enzymes** | **Fragment length** |
| --- | --- | --- | --- | --- | --- | --- | --- |
| **1.** | FTO | Forward primer | AACTGGCTCTTGAATGAAATAGGATTCAGA | Shahid et al., 2013 | Initial denaturation at 95°C for 4min, followed by 35 cycles of denaturation at 94°C for 30 sec, annealing at 58 °C for 30 sec and extension at 72°C for 1min, and then final extension at 72°C for 10 min and 4°C forever | *ScaI* | 182 bp, 154 bp and 28 bp |
|  |  | Reverse primer | AGAGTAACAGAGACTATCCAAGTGCAGTAC |  |  |  |  |
| **2.** | MC4R | Forward primer | AAGTTCTACCTACCATGTTCTTGG | Zlatohlavek et al., 2013 | Initial denaturation at 94^0^C for 4 minutes, followed by 35 cycles of denaturation at 95°C for 30 sec, annealing at 58 °C for 30 sec and extension at 72°C for 30 sec, and then final extension at 72°C for 5 min and 4°C forever | *Bcl II* | 107bp, 137 bp, and 30bp |
|  |  | Reverse primer | TTCCCCCTGAAGCTTTTCTTGTCATTTTGAT |  |  |  |  |
| **3.** | MTHFR | Forward primer | TGAAGGAGAAGGTGTCTGCGGGA | Frosst et al., 1995 | Initial denaturation at 94°C for 4min, followed by 35 cycles of denaturation at 94°C for 1 min, annealing at 63 °C for 1 min and extension at 72°C for 1min, and then final extension at 72°C for 7 min and 4°C forever | *Hinf I* | 198bp, 175bp and 23bp, |
|  |  | Reverse primer | AGGACGGGTGC GGTGAGAG |  |  |  |  |
| **4.** | ACE | Forward primer | CTG GAG ACC ACT CCC ATC CTT TCT | Rigat et al., 1990 | Initial denaturation at 94°C for 1 min, followed by 30 cycles of denaturation at 94°C for 1 min, annealing at 60 °C for 1 min and extension at 72°C for 1min, and then final extension at 72°C for 10 min and 4°C forever | - | 490bp, 190bp |
|  |  | Reverse primer | GAT GTG GCC ATC ACA TCC GTC AGAT |  |  |  |  |

Supplementary Table 2: General characteristics of cases and controls with respect to body mass index (BMI) among the Liangmai and Mizo tribes of Manipur

| Variables | | Liangmai  N (%) | | | Mizo  N (%) | | |
| --- | --- | --- | --- | --- | --- | --- | --- |
|  |  | Controls | Cases | χ^2^ p-value | Controls | Cases | χ^2^ p-value |
| Age, Median (IQR) | | 40 (30-48) | 42 (35-50) | < 0.07*^#^ | 36 (29-55) | 47 (36-56) | < 0.001*^#^ |
| Sex | Female | 119 (84.4) | 173 (81.6) | 0.49 | 79 (60.3) | 145 (53.1) | 0.17 |
|  | Male | 22 (15.6) | 39 (18.4) |  | 52 (39.7) | 128 (46.9) |  |
| Alcohol Consumption | No | 27 (72.9) | 28 (82.4) | 0.34 | 81 (61.8) | 185 (68.1) | 0.21 |
|  | Yes | 10 (27.1) | 6 (17.6) |  | 50 (38.2) | 87 (31.9) |  |
| Tobacco Consumption | No | 23 (62.2) | 28 (77.8) | 0.14 | 51 (38.9) | 81 (29.8) | 0.06 |
|  | Yes | 14 (37.8) | 8 (22.2) |  | 80 (61.1) | 191 (70.2) |  |
| Education | Literate | 111 (81.1) | 181 (85.8) | 0.23 | 131 (100) | 272 (100) | 1 |
|  | Illiterate | 26 (18.9) | 30 (14.2) |  | 0 | 0 |  |
| Occupation | Active | 116 (87.8) | 131 (68.2) | < 0.001 | 64 (48.8) | 134 (49.3) | 0.93 |
|  | Sedentary | 16 (12.2) | 61 (31.8) |  | 67 (51.2) | 138 (50.7) |  |

Cases-overweight/obese, Controls-normal/underweight; ^#^Mann-Whitney p-value; Yates’ corrected, N (%)-number; p-value<0.05 are statistically significant, p-value <0.09 and >0.05 are suggestive.

Supplementary Table 3: General characteristics of cases and controls with respect to waist circumference (WC) among the Liangmai and Mizo tribe of Manipur

| Variables | | Liangmai  N (%) | | | Mizo  N (%) | | |
| --- | --- | --- | --- | --- | --- | --- | --- |
|  |  | Controls | Cases | χ^2^ p-value | Controls | Cases | χ^2^ p-value |
| Age, Median (IQR) | | 41 (31-49) | 40 (32-50) | 0.85* | 36 (28-54) | 49 (38-56) | < 0.001* |
| Sex | Female | 90 (73.2) | 201 (88.5) | < 0.001 | 60 (40.8) | 165 (64.2) | < 0.001 |
|  | Male | 33 (26.8) | 26 (11.5) |  | 87 (59.2) | 92 (35.8) |  |
| Alcohol Consumption | No | 17 (68) | 37 (82.2) | 0.17 | 87 (59.2) | 180 (70) | 0.02 |
|  | Yes | 8 (32) | 8 (17.8) |  | 60 (40.8) | 77 (30) |  |
| Tobacco Consumption | No | 17 (68) | 30 (69.8) | 0.87 | 56 (38.1) | 77 (30) | 0.09 |
|  | Yes | 8 (32) | 13 (30.2) |  | 91 (61.9) | 180 (70) |  |
| Education | Literate | 97 (80.2) | 192 (85.7) | 0.18 | 147 (100) | 257 (100) | 1.00 |
|  | Illiterate | 24 (19.8) | 32 (14.3) |  | 0 (0) | 0 (0) |  |
| Occupation | Active | 95 (81.9) | 150 (73.2) | 0.07 | 67 (45.6) | 132 (51.4) | 0.26 |
|  | Sedentary | 21 (19.1) | 55 (26.8) |  | 80 (54.4) | 125 (48.6) |  |

Cases-high WC, Controls- normal WC; *Mann-Whitney p-value; Yates’ corrected, N (%)-number; p-value <0.05 are statistically significant.

Supplementary Table 4: General characteristics of cases and controls with respect to waist-hip ratio (WHR) among the Liangmai and Mizo tribe of Manipur

| Variables | | Liangmai  N (%) | | | Mizo  N (%) | | |
| --- | --- | --- | --- | --- | --- | --- | --- |
|  |  | Controls | Cases | χ^2^ p-value | Controls | Cases | χ^2^ p-value |
| Age, Median (IQR) | | 41 (32-50) | 40 (31-49) | 0.75^*^ | 35 (28-51) | 46 (35-57) | < 0.001^*^ |
| Sex | Female | 10 (37) | 281 (86.9) | < 0.001 | 19 (29.2) | 206 (60.8) | < 0.001 |
|  | Male | 17 (63) | 42 (13.1) |  | 46 (70.8) | 133 (39.2) |  |
| Alcohol Consumption | No | 2 (40) | 53 (81.5) | 0.02 | 35 (53.8) | 232 (68.4) | 0.02 |
|  | Yes | 3 (60) | 12 (18.5) |  | 30 (46.2) | 107 (31.6) |  |
| Tobacco Consumption | No | 1 (16.7) | 47 (74.6) | 0.003 | 21 (32.3) | 112 (33) | 0.90 |
|  | Yes | 5 (83.3) | 16 (25.4) |  | 44 (67.7) | 227 (67) |  |
| Education | Literate | 24 (88.8) | 266 (83.4) | 0.45^!^ | 65 (100) | 339 (100) | 1.00 |
|  | Illiterate | 3 (11.2) | 53 (16.6) |  | 0 | 0 |  |
| Occupation | Active | 19 (73.4) | 227 (76.7) | 0.67 | 23 (35.4) | 176 (51.9) | 0.01 |
|  | Sedentary | 7 (26.9) | 69 (23.3) |  | 42 (64.6) | 163 (48.1) |  |

Cases-high WHR, Controls- normal WHR; *Mann-Whitney p-value; Yates’ corrected, N (%)-number; p-value <0.05 are statistically significant.

Supplementary Table 5: General characteristics of cases and controls with respect to Waist Height Ratio (WHtR) among the Liangmai and Mizo tribes

| Variables | | Liangmai  N(%) | | | Mizo  N(%) | | |
| --- | --- | --- | --- | --- | --- | --- | --- |
|  |  | Controls | Cases | χ^2^ p-value | Controls | Cases | χ^2^ p-value |
| Age, Median (IQR) | | 41 (30-50) | 40 (32-49) | 0.04^*^ | 33.5 (27-44) | 49 (37-57) | < 0.001^*^ |
| Sex | Female | 56 (81.2) | 236 (83.4) | 0.65 | 51 (51) | 174 (57.2) | 0.27 |
|  | Male | 13 (18.8) | 47 (16.6) |  | 49 (49) | 130 (42.8) |  |
| Alcohol Consumption | No | 9 (64.3) | 46 (80.7) | 0.33^!^ | 54 (54) | 213 (70.1) | 0.003 |
|  | Yes | 5 (35.7) | 11 (19.3) |  | 46 (46) | 91 (29.9) |  |
| Tobacco Consumption | No | 8 (53.3) | 40 (72.7) | 0.26^!^ | 37 (37) | 96 (31.6) | 0.31 |
|  | Yes | 7 (46.7) | 15 (27.3) |  | 63 (63) | 208 (68.4) |  |
| Education | Literate | 51 (75) | 241 (86.1) | 0.02 | 100 (100) | 304 (100) | 1.00 |
|  | Illiterate | 17 (25) | 39 (13.9) |  | 0 (0) | 0 (0) |  |
| Occupation | Active | 60 (89.6) | 187 (72.8) | 0.004 | 43 (43) | 156 (51.3) | 0.14 |
|  | Sedentary | 7 (10.4) | 70 (27.2) |  | 57 (57) | 148 (48.7) |  |

Cases-high WHtR and controls- normal WHtR; *Mann-Whitney p-value; ! Yates’ corrected; N (%)-number; p-value <0.05 are statistically significant.

Supplementary Table 6: General characteristics of cases and control with respect to Total cholesterol (TC) among the Liangmai and Mizo tribes of Manipur.

| Variables | | Liangmai N (%) | | | Mizo N (%) | | |
| --- | --- | --- | --- | --- | --- | --- | --- |
|  |  | Controls | Cases | χ^2^ p-value | Controls | Cases | χ^2^ p-value |
| Age (Median) | | 40 | 43 | 0.048* | 45 | 47 | 0.527* |
| Sex | Female | 181 | 103 | 0.35 | 90 | 119 | 0.60 |
|  | Male | 42 | 18 |  | 65 | 96 |  |
| Alcohol  Consumption | No | 31 | 24 | 0.17 | 96 | 157 | 0.023 |
|  | Yes | 12 | 4 |  | 59 | 58 |  |
| Tobacco  Consumption | No | 29 | 19 | 0.45 | 37 | 95 | <0.001 |
|  | Yes | 14 | 6 |  | 118 | 120 |  |
| Education | Literate | 183 | 102 | 0.38 | 155 | 215 |  |
|  | Illiterate | 38 | 16 |  | 0 | 0 |  |
| Occupation | Active | 161 | 80 | 0.28 | 71 | 117 | 0.10 |
|  | Sedentary | 45 | 30 |  | 84 | 98 |  |

Cases-high TC and controls- normal TC; *Mann-Whitney p-value; Yates’ corrected; N (%)-number; p-value <0.05 are statistically significant

7

Supplementary Table 7: General characteristic of cases and controls with respect to the TG (Triglyceride) the Liangmai and Mizo Tribes of Manipur

| Variables | | Liangmai N (%) | | | Mizo N (%) | | |
| --- | --- | --- | --- | --- | --- | --- | --- |
|  |  | Controls | Cases | **χ^2^ p-value** | Controls | Cases | **χ^2^ p-value** |
| Age Median (IQR) | | 40 | 42 | 0.588* | 45 | 47 | 0.349* |
| Sex | Female | 176 | 108 | 0.44 | 114 | 96 | 0.08 |
|  | Male | 34 | 26 |  | 73 | 88 |  |
| Alcohol  Consumption | No | 29 | 26 | 0.48 | 122 | 131 | 0.24 |
|  | Yes | 10 | 6 |  | 64 | 53 |  |
| Tobacco  Consumption | No | 27 | 21 | 0.76 | 68 | 64 | 0.72 |
|  | Yes | 11 | 10 |  | 118 | 120 |  |
| Education | Literate | 176 | 110 | 0.94 | 186 | 184 |  |
|  | Illiterate | 33 | 21 |  | 0 | 0 |  |
| Occupation | Active | 146 | 95 | 0.90 | 97 | 91 | 0.60 |
|  | Sedentary | 46 | 29 |  | 89 | 93 |  |

Cases-high TG and controls- normal TG; *Mann-Whitney p-value; ^!^ Yates’ corrected; N(%)-number; p-value <0.05 are statistically significant

8

Supplementary Table 8: General characteristic of cases and controls with respect to the HDL among the Liangmai and Mizo tribes of Manipur

| Variables | | Liangmai N (%) | | | Mizo N (%) | | |
| --- | --- | --- | --- | --- | --- | --- | --- |
|  |  | Controls | Cases | **χ^2^ p-value** | Controls | Cases | **χ^2^ p-value** |
| Age Median (IQR) | | 40 | 41 | 0.378* | 44 | 51 | 0.012* |
| Sex | Female | 121 | 163 | <0.001 | 129 | 80 | <0.001 |
|  | Male | 45 | 15 |  | 134 | 27 |  |
| Alcohol  Consumption | No | 39 | 16 | 0.86 | 175 | 78 | 0.23 |
|  | Yes | 11 | 5 |  | 88 | 29 |  |
| Tobacco  Consumption | No | 34 | 14 | 0.64 | 105 | 27 | 0.007 |
|  | Yes | 16 | 5 |  | 158 | 80 |  |
| Education | Literate | 146 | 140 | 0.03 | 263 | 107 | - |
|  | Illiterate | 19 | 35 |  | 0 | 0 |  |
| Occupation | Active | 111 | 130 | 0.36 | 131 | 57 | 0.54 |
|  | Sedentary | 39 | 36 |  | 132 | 50 |  |

Cases-Low HDL and controls- normal HDL; *Mann-Whitney p-value; ^!^Yates’ corrected; N(%)-number; p-value <0.05 are statistically significant

9

Supplementary Table 9: General characteristic of cases and controls with respect to LDL among the Liangmai and Mizo tribes of Manipur

| Variables | | Liangmai N (%) | | | Mizo N (%) | | |
| --- | --- | --- | --- | --- | --- | --- | --- |
|  |  | Controls | Cases | **χ^2^ p-value** | Controls | Cases | **χ^2^ p-value** |
| Age Median (IQR) | | 40 | 43.5 | 0.009* | 45 | 48 | 0.467* |
| Sex | Female | 199 | 85 | 0.06 | 101 | 108 | 0.88 |
|  | Male | 49 | 11 |  | 79 | 82 |  |
| Alcohol  Consumption | No | 42 | 13 | 0.54 | 114 | 139 | 0.04 |
|  | Yes | 14 | 2 |  | 66 | 51 |  |
| Tobacco  Consumption | No | 36 | 12 | 0.25 | 44 | 88 | <0.001 |
|  | Yes | 19 | 2 |  | 136 | 102 |  |
| Education | Literate | 203 | 83 | 0.30 | 180 | 190 | - |
|  | Illiterate | 42 | 12 |  | 0 | 0 |  |
| Occupation | Active | 180 | 61 | 0.17 | 81 | 107 | 0.02 |
|  | Sedentary | 50 | 25 |  | 99 | 83 |  |

Cases-High LDL and controls- normal LDL; *Mann-Whitney p-value; ^!^Yates’ corrected; N(%)-number; p-value <0.05 are statistically significant

10

Supplementary Table 10: General characteristic of cases and controls with respect to VLDL among the Liangmai and Mizo tribes of Manipur

| Variables | | Liangmai N (%) | | | Mizo N (%) | | |
| --- | --- | --- | --- | --- | --- | --- | --- |
|  |  | Controls | Cases | χ^2^ p-value | Controls | Cases | χ^2^ p-value |
| Age (Median IQR) | | 40 | 42 | 0.588* | 45 | 47 | 0.349* |
| Sex | Female | 176 | 108 | 0.45 | 113 | 96 | 0.09 |
|  | Male | 34 | 26 |  | 73 | 88 |  |
| Alcohol  Consumption | No | 29 | 26 | 0.48 | 122 | 131 | 0.24 |
|  | Yes | 10 | 6 |  | 64 | 53 |  |
| Tobacco  Consumption | No | 27 | 21 | 0.76 | 69 | 64 | 0.67 |
|  | Yes | 11 | 10 |  | 118 | 120 |  |
| Education | Literate | 176 | 110 | 0.94 | 186 | 184 | - |
|  | Illiterate | 33 | 21 |  | 0 | 0 |  |
| Occupation | Active | 146 | 95 | 0.90 | 97 | 91 | 0.60 |
|  | Sedentary | 46 | 29 |  | 89 | 93 |  |

Cases-High VLDL and controls- normal VLDL; *Mann-Whitney p-value; ^!^Yates’ corrected; N(%)-number; p-value <0.05 are statistically significant

11
